# Bilateral Gastrocnemius Tertius Muscles: Cadaveric Findings of a Rare Variant

**DOI:** 10.7759/cureus.45316

**Published:** 2023-09-15

**Authors:** Isaac A Arefi, Ethan Kosco, Erica Werner, Aidan Maxwell, Andrew Fickert, Patrick W Frank

**Affiliations:** 1 Department of Medicine, University of Toledo College of Medicine and Life Sciences, Toledo, USA; 2 Department of Anatomy, Drexel University College of Medicine, Philadelphia, USA

**Keywords:** popliteal fossa, gross anatomy, dissection, cadaver, accessory soleus, nerve entrapment, gastrocnemius tertius

## Abstract

The posterior compartment of the leg typically contains three muscles in the superficial flexor group: the gastrocnemius, plantaris, and soleus. The gastrocnemius has medial and lateral heads (MH and LH) that originate from the medial and lateral condyles of the femur, respectively. However, a third head (TH) of the gastrocnemius, is a rare accessory muscle bundle of the gastrocnemius muscle that covers the surface of the popliteal fossa.

Bilateral THs of gastrocnemius were identified in a 67-year-old male during a routine educational cadaveric dissection. Both gastrocnemius TH muscles consisted of a superficial belly with distinct neurovasculature heads and originated from the lateral condyle of the femur and inserted into the Achilles tendon. To our knowledge, the co-existence of bilateral gastrocnemius TH muscles has only been reported once.

The male donor was found to exhibit an anatomical anomaly and could be clinically underdiagnosed due to its clinically silent nature and the lack of reports. Insight into the potential implications of bilateral and unilateral gastrocnemius TH and identification during clinical evaluation offers a path for future research to better identify and manage cases of gastrocnemius TH and its effects.

## Introduction

The gastrocnemius muscle forms the major bulk of the posterior compartment of the leg and is instrumental to walking and posture. During embryological development, the skeletal muscles are formed in the eighth week of gestation. The gastrocnemius muscles are derived from the paraxial mesoderm [1]. While both heads of the gastrocnemius muscle take origin from the capsule of the knee joint, the medial head (MH) of the muscle originates from the posterior surface of the medial condyle of the femur. The lateral head (LH) originates from a facet on the superior posterolateral surface of the lateral condyle of the femur. This biarticular muscle distally inserts into the posterior surface of the broad membranous Achilles tendon. This tendon descends over the posterior ankle joint and ultimately inserts into the middle part of the calcaneal tuberosity. The motor nerve supplying the two gastrocnemius heads is the tibial nerve, receiving fibers from the L5, S1, and S2 nerve roots. The posterior leg is cutaneously supplied by the L4-S2 nerve roots. The gastrocnemius is a powerful plantar flexor and knee flexor. Therefore, this muscle provides significant propulsive force in regard to gait mechanics.

## Case presentation

**Figure 1:**
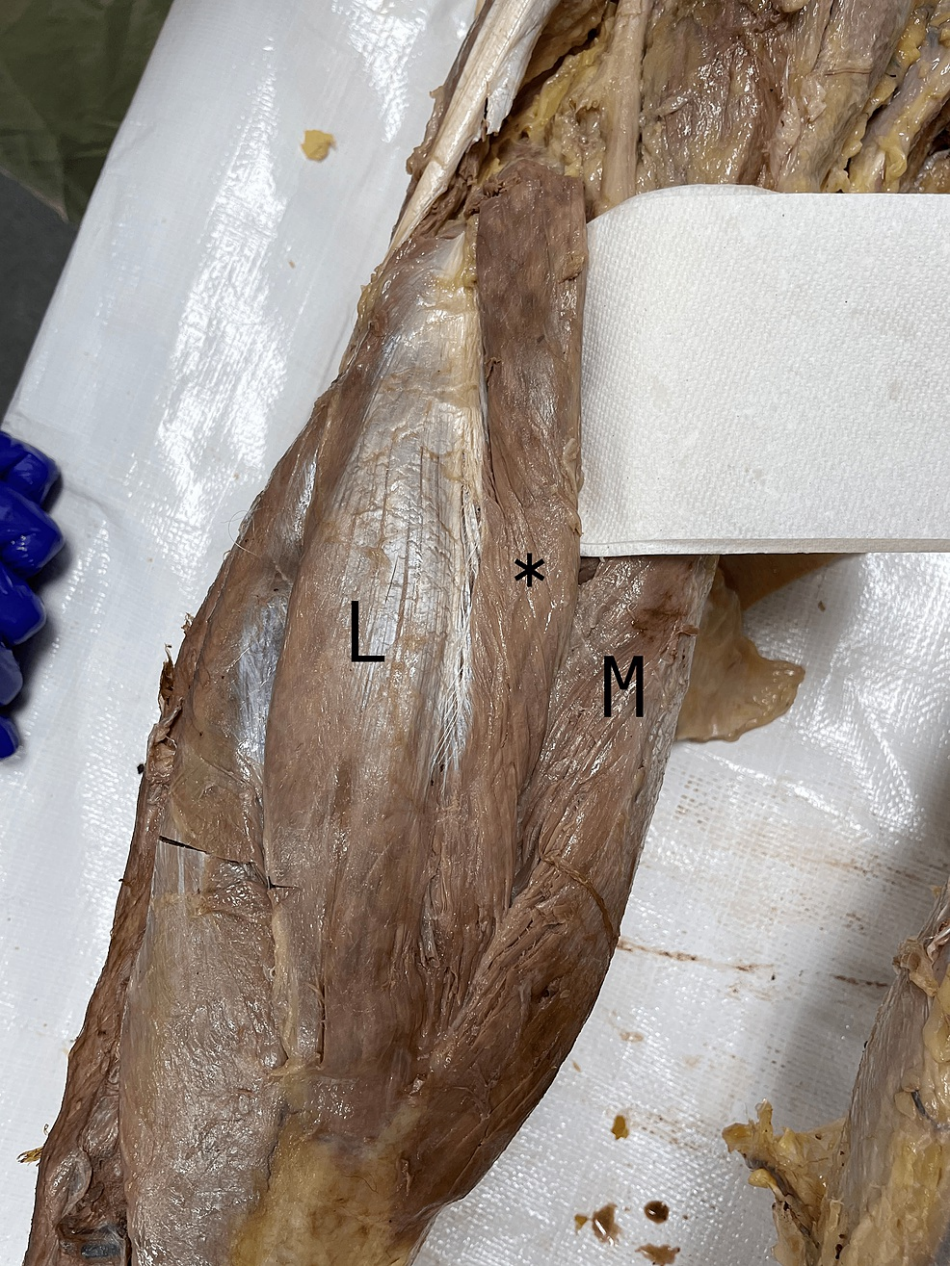
Photograph of the popliteal fossa of the left leg. Gastrocnemius third head (TH) is denoted by an asterisk (*), gastrocnemius medial head denoted by (M), gastrocnemius lateral head denoted by (L).

**Figure 2:**
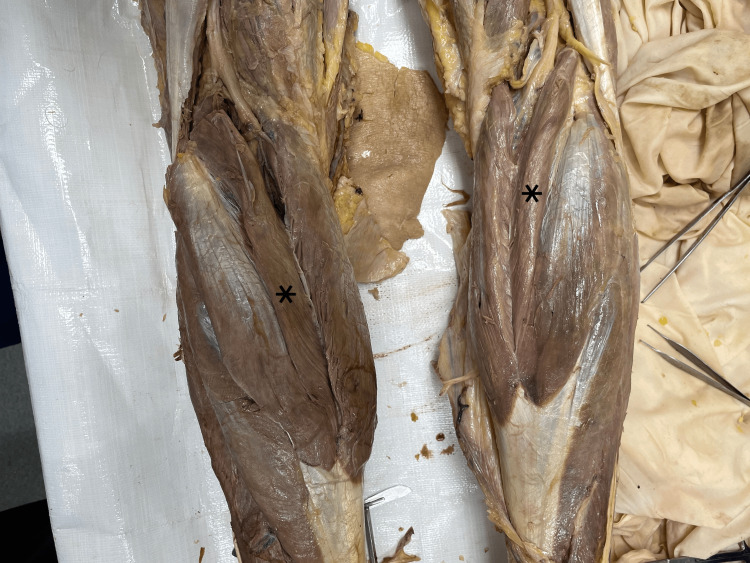
Photograph of the left and right popliteal fossa. Gastrocnemius third head (TH) is denoted by an asterisk (*).

**Figure 3:**
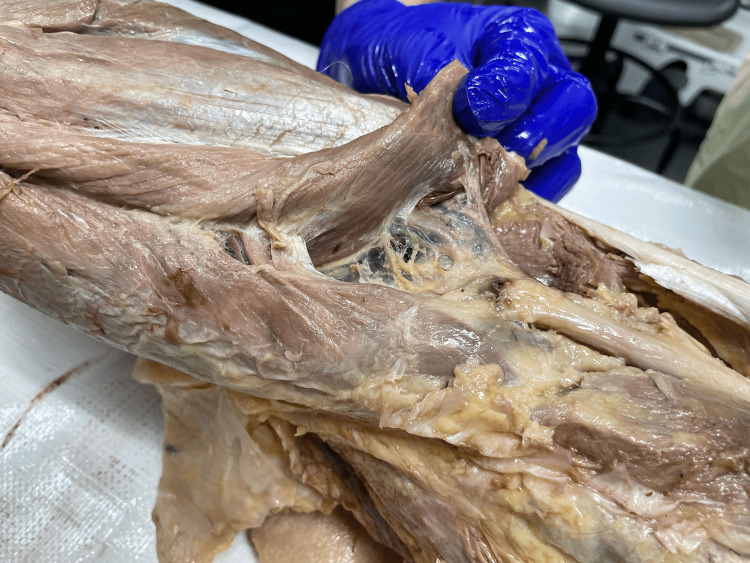
Photograph of popliteal fossa and neurovasculature of the left leg after incision and reflection of the gastrocnemius third head (TH).

**Figure 4:**
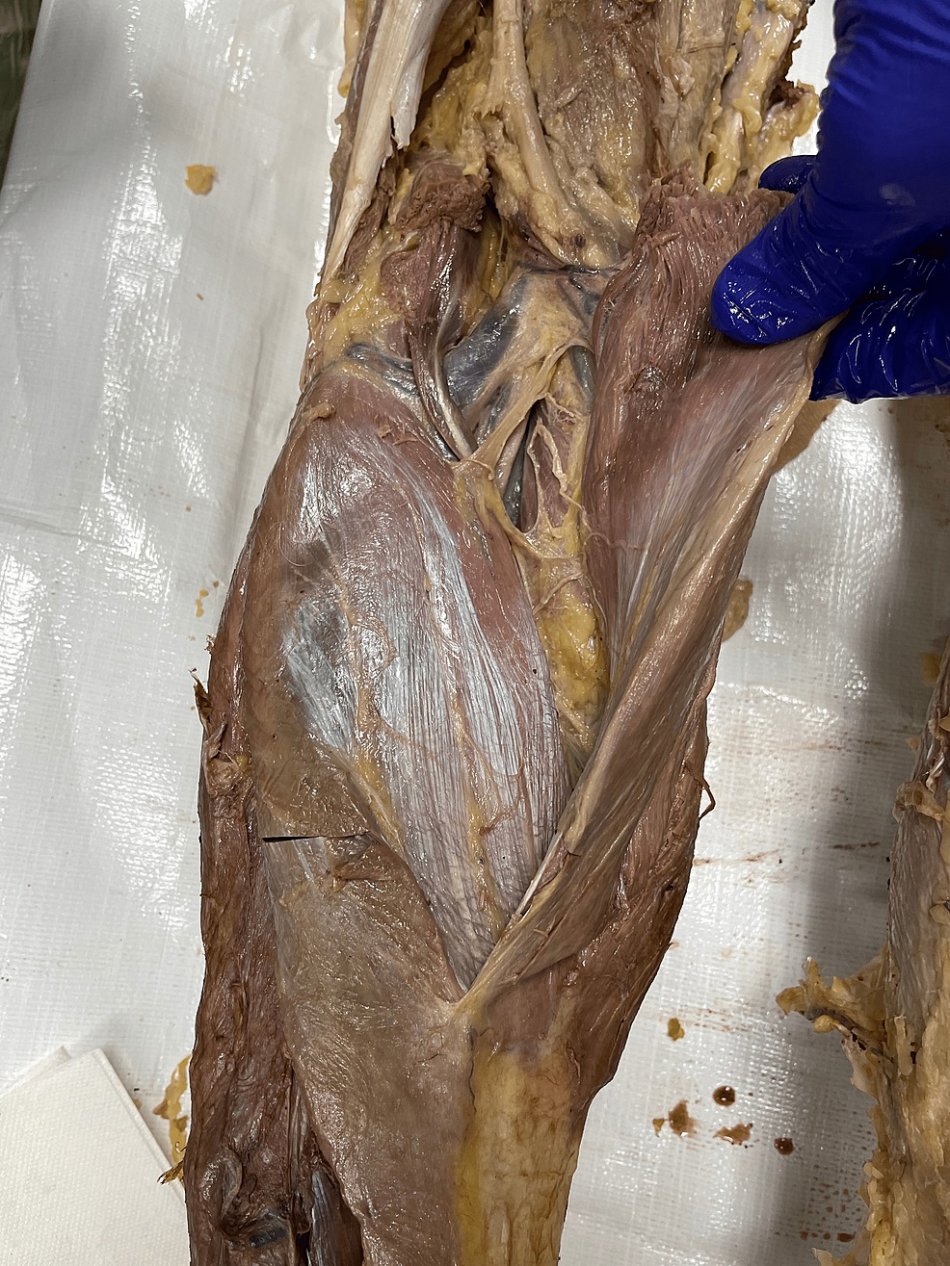
Photograph of popliteal fossa of the left leg after incision and reflection of the gastrocnemius lateral head and gastrocnemius third head.

This case features a 67-year-old man who graciously donated his body to the University of Toledo College of Medicine and Life Sciences donor program. The patient died from complications related to vascular dementia. All other details of his past medical history were not available due to the anonymity of the donor program. During cadaveric dissection, as part of a medical school curriculum, an extra muscle belly was discovered bilaterally, medial to each gastrocnemius LH; we have determined them to be TH of the gastrocnemius muscle. The THs were separated from the gastrocnemius LHs and MHs by a fascial sheath. Muscle fibers were arranged in a unipennate pattern. The THs attached proximally to the lateral condyle of the femur - at the same attachment point of the LH - and distally contributed to the Achilles tendon. The belly length was 24 cm, and the width was 2.5 cm. The TH muscles were innervated by a unique branch of the tibial nerve that entered the TH belly. Vasculature to the THs originated from additional branches of the popliteal artery and veins. The proximal tendon and belly of this muscle were in close proximity to the tibial nerve and artery. Structure and neurovasculature were consistent bilaterally and shown in Figures 1-4.

## Discussion

There are multiple case reports of the presence of a TH of gastrocnemius. However, to our knowledge, the occurrence of bilateral TH's has only been reported once before [2]. Our goal is to discuss this rare anatomical variant and propose the clinical manifestations as a result of this anomaly.

The presence of a TH of the gastrocnemius has been reported frequency of 1.9%-5.5% with varying levels of muscle belly size from threadlike to larger head in MRI studies. While most of these are reported to originate laterally within the posterior compartment, there was one report of the TH beginning medially within the posterior compartment [3]. Other than the fact that this can go undetected, the position of this extra muscle head may increase the risk and severity of certain conditions in individuals. One disease that has been implicated as being associated with extra heads of the gastrocnemius is popliteal artery entrapment syndrome (PAES). PAES is a rare vascular disease where muscles behind the knee compress the popliteal artery. Reports have suggested that increased size and number of gastrocnemius muscle bellies leads to a higher incidence of popliteal nerve entrapment and incidence of deep vein thrombosis (DVT) [4]. It is uncertain whether this anatomical variation had an impact on this patient’s death of vascular dementia. Therefore, understanding the complex relation between this anatomical variation and resulting clinical outcomes is imperative. This results in vascular compromise within the popliteal fossa leading to pain with exertion [5]. The size of the TH varies from case to case; the correlation of larger gastrocnemius heads tends to cause more complications due to compression in local areas and vascular complications such as thromboembolism and ischemia [6].

Another implication is sural nerve entrapment. The sural nerve is a peripheral sensory nerve branch of the tibial nerve that provides sensation to the posterolateral aspect of the distal third of the leg and the foot and ankle. Thus, entrapment causes loss of sensation and numbness to the lateral leg and lateral malleolus due to gastrocnemius head size compressing the sural nerve [7]. Although traditionally believed to be a pure sensory nerve, some evidence suggests that the sural nerve also has a motor function [8]. Therefore, any entrapment of the sural nerve may have motor and sensory consequences for the lower leg muscles. On the contrary, we hypothesize that there may be increased power output from the additional head of the gastrocnemius muscle assuming no nerve or artery entrapment occurs. This would be in relation to the general difference in the size of the leg compared to a population without TH muscle presence. Additional research is also warranted for the possibility of exacerbation of nerve and artery entrapment through increased activity and underlying genetic correlation with the bilateral presence. We would like to speculate an increase in the success of gastrocnemius reconstruction in patients with dorsal leg trauma. 

With the above clinical implication in mind, if a physician notes an extra head of the gastrocnemius is present, they may be inclined to intervene depending on the patient's relevant history and future risk. With PAES and sural nerve entrapment as potential negative outcomes, surgical resection of the TH may be implicated. Functional MRI can be used to detect these syndromes and to reduce the risk of later vascular comorbidities [9]. There are many routes to discover the causes and outcomes of TH of gastrocnemius. Further research could allow clinicians to more accurately diagnose patients leading to more optimal patient outcomes.

## Conclusions

This case reports the rare presentation of bilateral TH muscles. Although believed to be a benign anatomical variant, this can potentially lead to pathologies such as DVT and PAES. Compression of these structures by the TH may cause numbness, tingling, swelling, and signs of muscle hypoxia in the posterior leg. Ultrasound, computed tomography (CT), or magnetic resonance angiography (MRA) can detect compression and warrant management in the form of resection or avoidance of strenuous lower body exercise. Therefore, clinicians should be aware of this variant and its clinical relevance. TH-related posterior leg vessel compression should be considered a differential when treating patients with signs and symptoms of lower leg swelling or pain. We recommend that gait-lab analysis be performed on patients with TH to study its impact on movement and biomechanics.
